# Effects of Landscape Structure on Medicinal Plant Richness in Home Gardens: Evidence for the Environmental Scarcity Compensation Hypothesis

**DOI:** 10.1007/s12231-018-9417-3

**Published:** 2018-05-30

**Authors:** Monika Kujawska, Fernando Zamudio, Lía Montti, Veronica Piriz Carrillo

**Affiliations:** 1 Institute of Ethnology and Cultural Anthropology, University of Lodz, ul. Lindleya 3/5, 90-131 Lodz, Poland; 2 Interacciones ecológicas y conservación, Instituto Multidisciplinario de Biología Vegetal (CONICET-UNC), Av. Vélez Sarsfield 299, 5000 Córdoba, Argentina; 3 Instituto de Investigaciones Marinas y Costeras, UNMdP-CONICET & Instituto de Geología de Costas y del Cuaternario, UNMdP-CIC, Deán Funes 3350-CP 722, Buenos Aires, Argentina; 4 Instituto de Biología Subtropical, UNaM-CONICET, Bertoni 85, CP 3370, Puerto Iguazú, Misiones Argentina; 5 Tucuman, Argentina

**Keywords:** Agrobiodiversity, Domestication, Ethnobotany, Geospatial tools, Land use, Migrants, Paraguayan farmers, Plant management, Atlantic Forest

## Abstract

Our research involves of how Paraguayan migrants who are living in Misiones, Argentina, manage medicinal plants in home gardens, and how this practice can be related to the landscape. We examine the relationship between the richness of home garden medicinal plants and landscape variables (e.g., distance to the forest) by applying PLS analysis, which combines principal component analysis with linear regression. We surveyed 60 home gardens localized in a rural area, and we characterized the surrounding landscape with geospatial tools. Paraguayans’ home gardens are extremely diverse sites (total of 136 medicinal species), where both native (82) and introduced species (50) are managed. People who live close to the native forest or mixed use areas (e.g., farms, secondary vegetation) tend to possess less native plants in their gardens because they are available nearby. While gardeners, who live in proximity to tree crops (e.g., pine plantations), have reduced access to wild medicinal resources; therefore, their effort is concentrated on maintaining native plants. These results reflect a relationship between accessibility to medicinal plants in the landscape and the management practices in the home gardens, a neglected driver in explaining the richness and composition of the medicinal plants in home gardens so far. Thus, we contributed evidence in support of the environmental scarcity compensation hypothesis. Finally, our study supports the idea that home gardens appear to function as a springboard for plant domestication.

**Electronic supplementary material:**

The online version of this article (10.1007/s12231-018-9417-3) contains supplementary material, which is available to authorized users.

## Introduction

Over the last few decades, there has been a surge of home garden studies utilizing diverse methodological approaches and conceptual frameworks. Scholars have formulated questions related to plant use, richness, diversity, composition, and structure within these social-productive units (Padoch and de Jong 1991; Parihaar et al. 2014; Smith et al. 2006; Sujarwo and Caneva 2015). It has been acknowledged that both intrinsic and extrinsic factors affect the richness of species in home gardens. Common intrinsic factors are, for example, the size of the garden, its age, and its proximity to the dwelling, among others (Blanckaert et al. 2004; Coomes and Ban 2004; Sander and Vandebroek 2016). On the other hand, research indicates that factors extrinsic to the garden, such as distance to urban centers, kinship, size of family groups, forms of management, and networks of exchange, may explain the species richness and diversity of home gardens (Díaz-Reviriego et al. 2016; Ellen and Platten 2011; Lamont et al. 1999; Peroni et al. 2016; Sander and Vandebroek 2016). For example, studies have shown that peri-urban and urban home gardens may host a greater diversity of species than those situated in remote places. This is explained by wider opportunities for knowledge and plant exchange, especially in intercultural contexts (Bernholt et al. 2009; Heckler 2007; WinklerPrins 2002). More recent studies, however, have demonstrated that biodiversity in home gardens differentiates along the rural-peri-urban gradient, without necessarily increasing or declining (Peroni et al. 2016; Poot-Pool et al. 2015). While the richness of useful tree and shrub species, mainly native ones, tends to decrease, the number of herbaceous species, especially introduced ornamental ones, increases in urban space (Poot-Pool et al. 2015).

Home gardens are one of the oldest forms of land use (Kumar and Nair 2004). They represent reservoirs of currently cultivated and potentially useful resources (Alvarez-Buylla et al. 1989), and provide a wide range of ecosystem services (Calvet-Mir et al. 2012). Importantly, there is a considerable consensus in favor of acknowledging rural and urban gardeners for their in situ preservation of biological and genetic diversity (Galuzzi et al. 2010; Heraty and Ellstrand 2016; Kumar and Nair 2006; Saleko et al. 2014). Moreover, home gardens are suitable places for the study of gardeners’ choices concerning the selection of plant species for protection or even cultivation, and are very interesting sites for research on plant domestication (Blanckaert et al. 2004; Casas et al. 1996; Smith 1996).

As some studies suggest, a large proportion of native plants are moved to and cared for in domestic gardens (Blanckaert et al. 2004; Larios et al. 2013; Peroni et al. 2016). In addition, a remarkable proportion of spontaneously growing plants, especially medicinally useful ones, are spared by gardeners (Blanckaert et al. 2004). This is explained by the strong interaction between home garden plants and the surrounding vegetation (Blanckaert et al. 2004). While there are studies analyzing the relationship between landscape and human accessibility, such as distance from human settlements and plant diversity in the local landscape (see Thomas et al. 2009), little attention has been paid so far to the relationship between the structure and composition of the surrounding landscape and the diversity and management of home gardens. Haines-Young (2009) affirms that understanding of the relationships between land use and biodiversity is fundamental to understanding the links between people and their environment, while the way in which land is managed are key drivers of changes in biodiversity. Hence, changes in land use influence the structure, function, and dynamics of socio-ecological systems (Orozco et al. 2015).

Access to medicinal resources may be influenced by the structure, composition and distribution of native vegetation, productive activities related to land use (agriculture, forestry, etc.), the presence of roads, the intensity of use of these resources, and the requirements and habits of medicinal species. Deforestation itself affects access to these species by decreasing availability (see Shanley and Luz 2003). While the debate about the relative contribution of primary and secondary vegetation to the acquisition of medicinal plants persists, evidence backs up the hypothesis that disturbed vegetation may constitute a preferred habitat for collectors and users of medicinal plants (Gavin 2009; Voeks 1996). Moreover, Gavin (2009) estimates that landscape that includes forests of different ages can maximize the availability of medicinal plant species. However, the effect of different vegetation types of landscape on the diversity of home gardens is an emerging focus. The only information available in this regard has been outlined by Larios and collaborators (2013), who found that the highest diversity was recorded in home gardens where the neighboring forest had the least diversity and vice versa. The authors conclude that local people mainly use their home gardens to manage plant species that are not available in the wilderness close to their towns. This phenomenon has been named the “scarcity compensation effect” (see Larios et al. 2013).

Our research has evolved around the two dominant questions of how local people manage plants in their home gardens and how this practice can be related to the surrounding landscape structure. Additionally, we would like to ask whether home gardens may become a convenient venue for the domestication of native medicinal plants and the pre-adaption or acclimation of potentially invasive alien species (see Marco et al. 2008).

In this paper, we propose an exploratory approach to the integration of ethnobiological research and geospatial tools in order to study access to medicinal plants by means of analysis of the landscape around home gardens. We worked in the home gardens of Paraguayan migrants who live in the Misiones province in Argentina.

Our hypothesis is presented as follows: given that Paraguayan migrants live in a landscape that is socio-productively homogenous on a large scale, they cultivate and use a similar diversity of medicinal species in their home gardens. However, the landscape structure close to home gardens influences the richness and status of medicinal plants in them, due to access to patches of different types of vegetation, from which plant material is obtained.

Our prediction is that home gardens surrounded by a continuous area of native forest or mixed use areas (i.e., small farms with a mosaic of subsistence crops, secondary vegetation, pastures and patches of degraded native forest), or located inside a heterogeneous landscape, have less richness of native medicinally useful species in their home gardens compared to home gardens located in the vicinity of productive forests of introduced species (e.g., *Pinus* spp.), due to the scarcity compensation effect. Thus people living in areas with a high proportion of native forest and mixed use vegetation, or with easy access to them, can find their native plant resources in nearby areas instead of having to cultivate or protect them in their home gardens. On the other hand, we do not expect a clear pattern concerning the richness of introduced species, because these are previously acclimatized and domesticated plants, easily available due to their widespread use in the region, and which are chiefly obtained through social networks, not from the landscape (Furlan et al. 2016).

Therefore, our objectives are to (1) evaluate the richness, status (native versus introduced) and management (cultivated, protected, transplanted, etc.) of medicinal plants found in the gardens of the Paraguayan immigrants in Misiones province; (2) describe the structure and characteristics of the nearby landscape where the home gardens of Paraguayan migrants are found; (3) evaluate the potential relationship between the richness of native and introduced medicinal species tended in home gardens and landscape variables (e.g., distance to the native forest).

## Methods

### Study Area

Our study area is the Misiones province located in the Argentinean Northeast. It is comprised of extensive native forest cover, next to former forest land currently used by companies (pine monocultures and processing factories), as well as medium-size cash crop production (*Ilex paraguariensis* A. St -Hil., *Citrus* spp. or *Camellia* spp.) complemented with animal husbandry (Hilgert et al. 2016; Mastrangelo et al. 2011).

The native cover of the total study area is semi-deciduous Atlantic Forest. The climate is subtropical humid with no dry seasons, a mean annual rainfall of 2000 mm and a mean annual temperature of 20 °C (Campanello et al. 2009). The forest is characterized by the presence of three well-defined canopy strata including more than 70 tree species, usually covered with numerous lianas and epiphytes, and mixed with shrubs, bamboos, and grasses (Campanello et al. 2009; Chediack 2008). Currently nearly 40% of the original forest cover is preserved in Argentina, in contrast to Brazil and Paraguay, where it comprises approximately 8% (Galindo-Leal and Câmara 2003). However, native forest cover is decreasing, due to an increasing population size in the region and the increasing demand for wood products coupled with the reduced economic value of the remaining forests—the result of uncontrolled selective logging (Campanello et al. 2009). Forest plantations, mainly *Pinus* and *Eucalyptus* species, have grown in area from 80,000 to 370,000 ha, an increase of 1 to 11% of the provincial territory between 1973 and 2006 (Izquierdo et al. 2008). These forest plantations are implemented and managed by large forestry companies and to a lesser extent by small and medium producers (Hilgert et al. 2016). Concurrently, the remaining natural forest persists principally within protected and uncultivated areas (e.g., high slopes or bad quality soil). The native vegetation also persists as degraded primary forest (*capueras*) and secondary forest alongside the farmland (Holz et al. 2009).

### Ethnographic Setting and Data Collection

Since the 19th century, mestizos (*criollos*) have been coming to Misiones province from the south of Argentina (Corrientes) and from neighboring Brazil and Paraguay. Since 1947, Paraguayans have been the most populous migrant community in Argentina. As of 2001, they have accounted for approximately 550,713 people, representing 30.5% of all immigrants in the country, and nearly 8.6% of the total population of Paraguay (INDEC 2010; Torres 2014). The push and pull factors for migration have been of both economic and political character (i.e., the Chaco War in 1936, the Paraguayan Civil War in 1947, and long years of military dictatorship ending in 1989), leading Paraguayan people to seek agricultural employment opportunities in the production of cotton, sugar cane, tea, and tobacco in the Argentinian northeast (Benencia 2007). Paraguayan migrants exhibit significant cultural cohesion in the study area, unified by language—most speak the native Guarani, intermarry, and have cultural institutions that keep them together (e.g., groupings of family residences, cultural associations, e.g., in Puerto Piray).

A total of 60 home gardens were surveyed along the study area. Home gardens are understood here as a front and backyard (Spanish: *el patio y el huerto*) adjacent to a home. In the case of Paraguayan migrants’ home gardens, the backyard can be partly or fully fenced, with some herbs (e.g., oregano, chive) and vegetables cultivated in it. Trees, ornamental, and medicinal plants usually grow in both the front and backyard (based on field observations).

Home garden inventories of medicinal plants were performed among 44 women (aged 30–95) and 16 men (aged 45–90). Adults participated voluntarily, recruited by snowball technique. Forty-three of the study participants hail from the rural areas of Eastern Paraguay, which belongs to the same ecoregion of the Atlantic Forest, while 17 were born in Argentina. Their parents were of Paraguayan origin and spoke Guarani, and all but two could speak Spanish. Fifty-two participants (87%) declared not having completed primary school, while only six have completed primary and two have completed secondary school. Forty-three participants claimed to have health insurance, while 17 declared that they did not have any. In general terms, the study participants can be described as small scale farmers working on 1–2 ha plots (*chacras*), where they cultivate staple crops such as manioc and maize, often intercropped with peanuts. Additionally, male participants usually find off-farm employment, mainly in the forest industry.

In most cases, the first author collected medicinal plants in the company of study participants. All of the documented botanical taxa are supported with herbarium specimens identified by the first author and stored in the CITS herbarium in the Instituto de Botánica del Nordeste (IBONE), Corrientes province, Argentina. Prior to each interview, study participants gave oral informed consent for their participation. Although there were no requirements for the project to pass any ethical commission, either in Argentinean or Polish institutions, the first author obtained permission from the Ministry of Ecology in Posadas, capital of Misiones, to collect herbarium specimens and for their transfer to the herbarium in Corrientes.

### Selection of Study Sites and Landscape Analysis

**Fig. 1:**
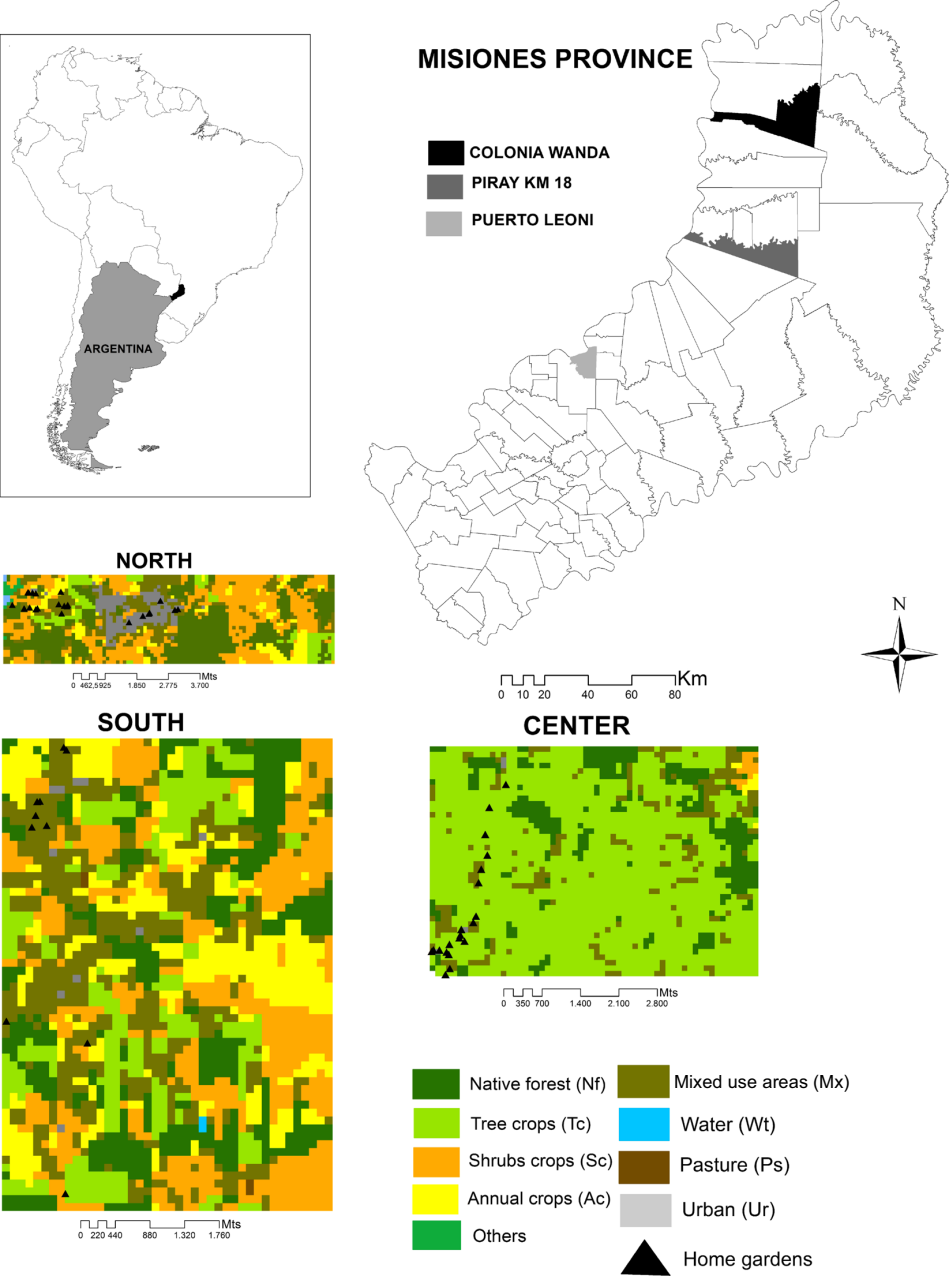
The greater study area in Misiones Province, Northeast of Argentina. In detail, the three study sites of the home gardens, each 25 km^2^ surface area showing the surrounding sampled land cover.

In order to achieve the greatest variability of the Paraguayans’ home gardens while also concentrating on work-sampling effort, we worked in and around three sites along the coast of the Parana River where Paraguayan migrants live (Fig. 1): (1) North of the study area within and around the municipal town of Colonia Wanda; (2) Center of the study area, called Piray Km 18, within the Puerto Piray municipality, which is a small settlement composed of farmhouses and small cultivated plots, and (3) South of the study area, near Puerto Leoni, a municipal town, the smallest of the three localities. These three sites are representative of the socio-productive landscape of the area and have a high concentration of Paraguayan migrants. The home gardens are located in a rural and semi-rural environment, near small towns, which have populations ranging from 2600 to 15,911 inhabitants (INDEC 2010).

In order to evaluate and quantify the landscape structure that surrounds the study home gardens, we used the land cover map of Misiones created by Zuleta et al. (2015). Land covers were re-classified into 10 classes: (1) *Urban* (Ur = town and other built-up areas), (2) *Native Forest* (Nf = native forest), (3) *Tree crops* (Tc = *Pinus* spp., *Eucalyptus* spp.), (4) *Shrub crops* (Sc = *Ilex paraguarensis*—yerba mate, *Citrus* spp., *Camellia* spp.—tea, and other perennial crops), (5) *Annual crops* (Ac = *Glycine max* (L.) Merr.—soybean, *Zea mays* L.—corn, and other herbaceous crop species), (6) *Mixed use areas* (Mx = small farms with a mosaic of subsistence crops, pasture, secondary vegetation, and patches of degraded native forest, (7) *Wetland* (Wl), (8) *Pasture* (Ps), (9) *Water bodies* (Wt = rivers, streams, lagoons and artificial water bodies), and (10) *Others* (natural deforested areas, bare soil, and not classified pixels).

Local people in the study area obtain medicinal plants from different vegetation units. Some of the species are exclusively obtained from the Native Forest (Keller 2008; Kujawska and Pardo de Santayana 2015), although mixed use vegetation has been found to be a site where many ruderal species of medicinal interest are harvested (Kujawska et al. 2012). Medicinal plants are also collected from farmland where perennial shrubs and annual crops are cultivated, but in lesser proportion than from forest and ruderal areas, due to use of pesticides and weeding practices (Kujawska and Pardo de Santayana 2015). Only a very limited number of medicinal species, e.g., *Nasturtium officinale* R.Br. grow in the wetlands. The spontaneous vegetation of the tree crops areas contributes to the least favorable conditions for the growth of medicinal species as it is the product of intensive management with the use of agrochemicals. As a result, the soils are extremely acidic by the deposition of acyclics, and this is also a very shady environment (Frank and Finckh 1997).

Beyond this classification, we characterized the landscape structure into three surface samples of 25 km^2^ (one per locality), including all home gardens (Fig. 1). A study of landscape features and computed descriptive statistics was conducted using ArcGis 10.1, Spatial Analysis tools and FRAGSTATS software (McGarigal and Marks 1995). The selection of metrics was based on their ability to characterize various aspects of fragmentation such as total area of fragments per class, mean size of patch (index of fragmentation), number of patches (indicator of several ecological processes and landscape heterogeneity), total edge (indicator of patch complexity), average nearest-neighbor distance and mean proximity index (see McGarigal et al. 2002).

### Data Analysis

**Fig. 2:**
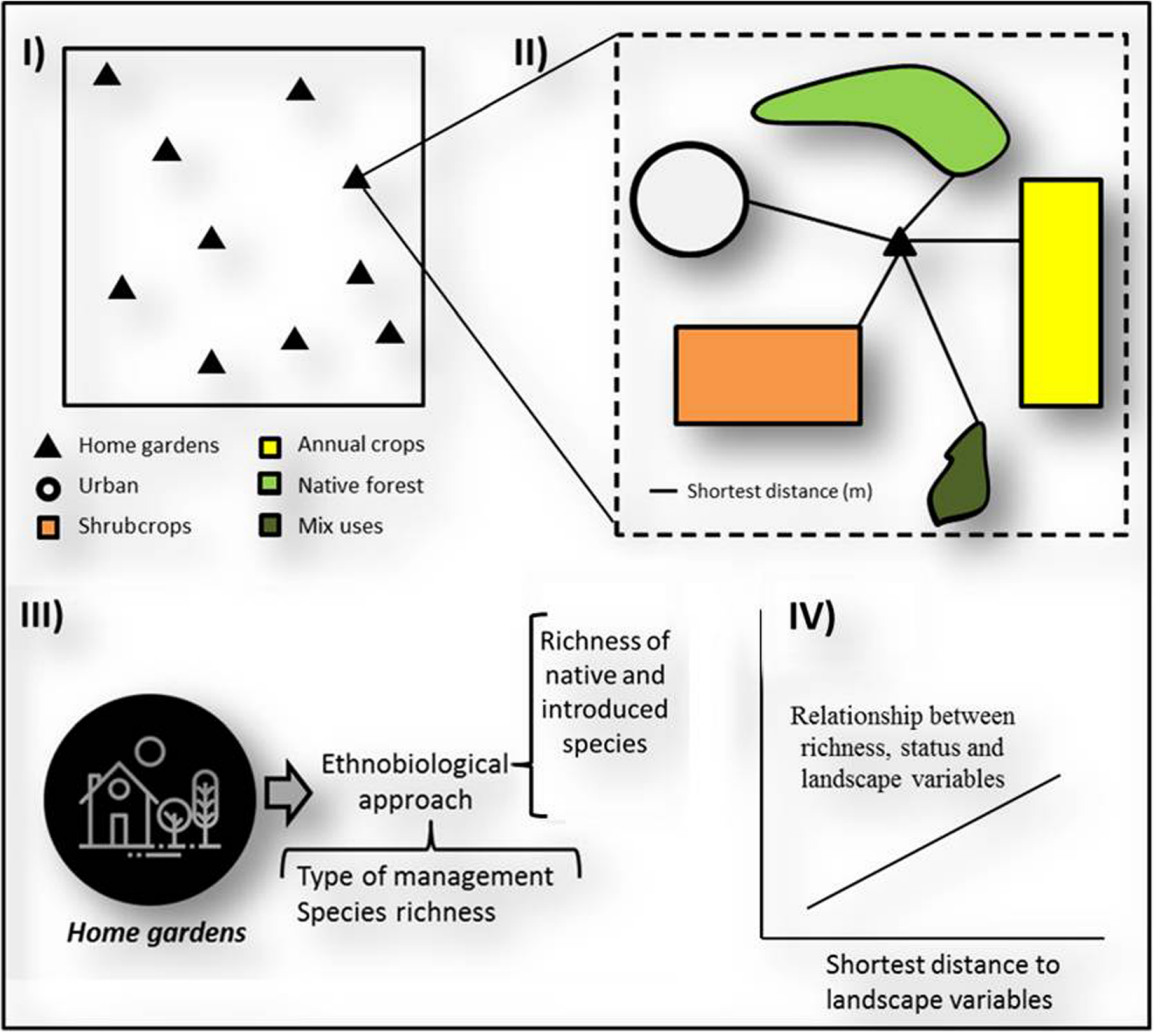
Synthesis of methods used in the analysis of the environmental scarcity effect.

In order to test the main hypotheses, we explored the relationship between the landscape variables (i.e., distance to different types of vegetation where medicinal plants can be found) and the richness of native and introduced species found in home gardens. Figure 2 shows a schematic representation of our methods and a conceptual framework of our methodological proposal. Using the multiple minimum distance tools from ArcGis 10.1, we calculated the shortest distance between home gardens and different land-use classes (i.e., Ur, Nf, Tc, etc.). We measured distance between each home garden and different vegetation classes except Wetland, Pasture, and Water bodies because these are poorly represented in the region and we used a new distance variable called Accr (access to national and/or provincial routes and secondary paved roads) to perform an independent analysis.

To evaluate the influence of factors associated with the landscape on the richness of native and introduced species in the home gardens, a partial least squares regression (PLS) was performed. This analysis is particularly appropriate when several predictors are correlated with each other and the number of cases is relatively low (Haelein and Kaplan 2004). PLS, which generalizes and combines principal component analysis with linear regression, consists in finding axes or latent variables that maximize the variation of the matrix of dependent variables, associated with the variation of the matrix of predictor variables. After generating latent variables with greater explanatory power, simple linear regressions were performed between the latent variables and the six predictor variables of the model. This was done in order to evaluate the degree of relationship between these latent variables and predictor variables, and thus, we were able to identify the variables that best explain the variation in the richness of native and introduced species of Paraguayans’ home gardens. All analyses were performed using the Infostat/P v2010 program (Di Rienzo et al. 2010).

To evaluate the management and the role of home gardens as social ecological spaces where the processes of domestication of native plants may occur, we performed a classification of the species according to the type of acquisition management that they were subjected to (Casas et al. 1996). Consequently, we divided the plant management into two stages: initial management and further management. In the first stage, we distinguished practices, such as planting, sowing, and direct transplant from other habitats. In the second stage, we distinguished two major practices, namely, cultivation and protection (see: Blanckaert et al. 2004; Casas et al. 1996; Lins Neto et al. 2014).

In this study, we used the *etic* notion of native and introduced species. We did not consider the perception of resources as familiar or new by study participants incorporated within living memory (see Heckler 2007; Peroni et al. 2016). Therefore, introduced plants are exotic species to the local flora, which arrived in Argentina with or without the help of people from the area. The list of native and introduced species was elaborated based on the *Flora de Conosur* (http://www.darwin.edu.ar/Proyectos/FloraArgentina/Familias.asp). With this analysis, we wanted to see whether migrants originating from a similar environment (i.e., Paraguay) contribute to the maintenance of diversity of native species in the host country (i.e., Argentina).

## Results

### Richness and the Status of Medicinal Plants in Home Gardens

**Fig. 3:**
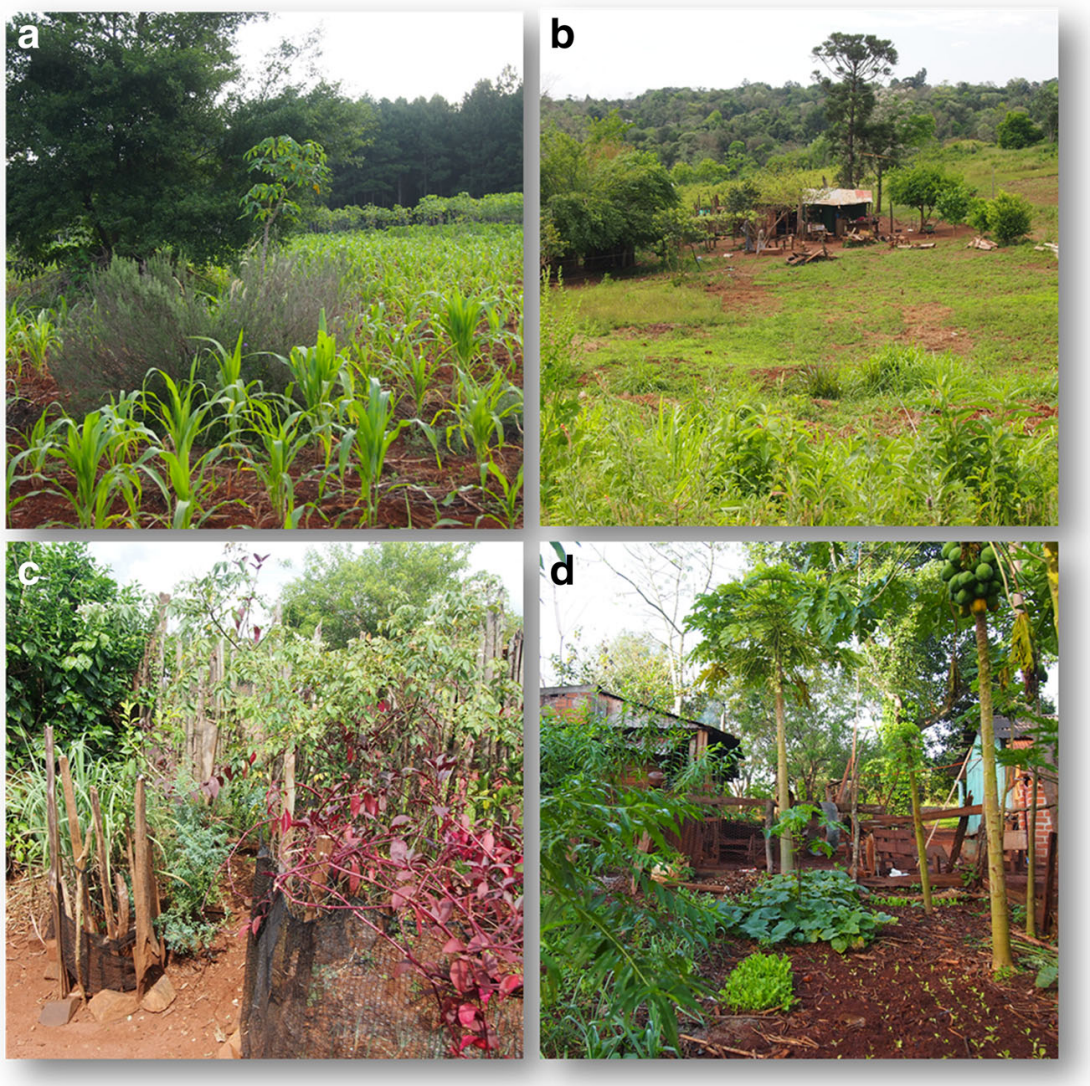
Examples of Paraguayan farmers’ home gardens and landscape, in which they are inserted. **a** A view on maize and cassava field and pine productive forest from fenced part of a home garden in Piray Km 18. **b** Household surroundings in Puerto Wanda. Fruit trees and other tree species are in the home garden. **c** Medicinal plant corner in a home garden of Puerto Wanda, Misiones: *Cymbopogon citratus, Ruta chalepensis, Rosa* sp., *Alternanthera brasiliana*. **d** Part of a home garden with edible and medicinal plants in Puerto Leoni, Misiones.

The overall richness of medicinal plants found in 60 Paraguayan home gardens corresponds to a total of 136 species with an average of 11.1 per home garden, the maximum being 24 and the minimum being 3 species (Appendix 1, Electronic Supplementary Material, ESM). The native taxa (82) outnumber the introduced ones (50). Four naturalized species are cultivated, with an average of 0.2 species per home garden. Native and introduced species are equally represented when analyzing the average per home garden (5.4 and 5.6, respectively). The home gardens that have more introduced species than natives represent 47% of the total, while those that have more native than introduced species, or the same proportion, reach 35 and 11%, respectively. Notably, gardens with more native species present a proportion up to three times greater than those that have a prevalence of introduced species. However, these are not necessarily the most diverse gardens. For example, a home garden with 5 native and 1 introduced species equals a proportion native/introduced of 5 (Fig. 3).

The species with the highest frequency of occurrence was the native *Lippia alba* (Mill.) N.E.Br. ex Britton & P.Wilson and was present in 32 home gardens, accounting for more than 50% of all the studied home gardens. However, in general terms, introduced species exhibit greater frequency of occurrence than natives, with six species present in at least 1/3 of home gardens: *Aloe maculata* All., *Cymbopogon citratus* (DC.) Stapf, *Ruta chalepensis* L., *Rosmarinus officinalis* L., *Artemisia absinthium* L., and *Mentha* spp. Among the naturalized plants, *Citrus* x *aurantium* L. was mentioned with the greatest frequency, but it was found in only six gardens (Appendix 1, ESM).

### Landscape Structure

**Fig. 4:**
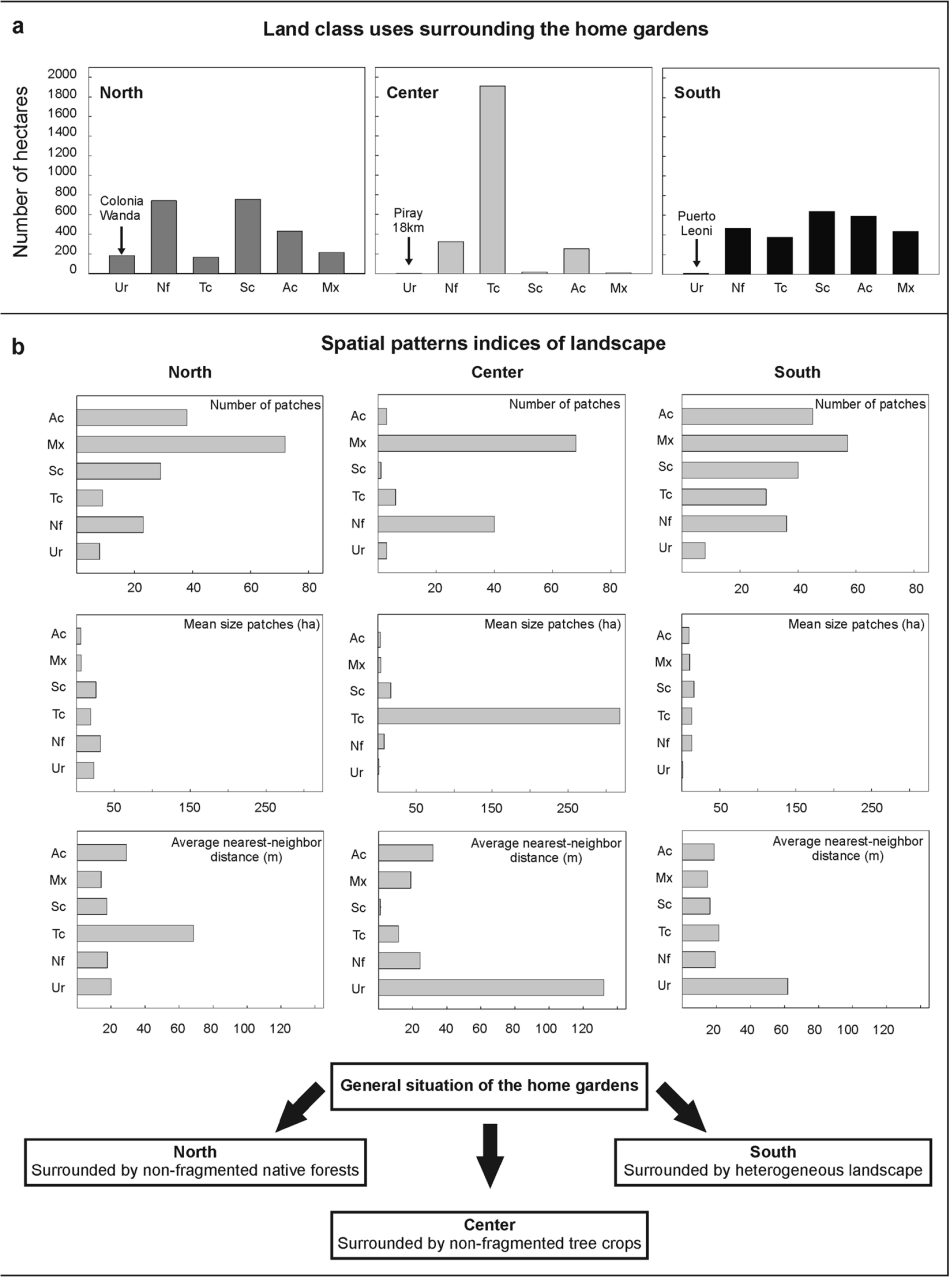
**a** Land class uses surrounding the home gardens in the three surveyed areas. **b** Different indices of spatial patterns of landscape. Bars indicate cover use classes. Ur (Urban area); Nf (Native forest); Tc (Tree crops); Sc (Shrubs crops); Ac (Annual crops); Mx (Mixed use area). Wetlands and Pasture classes were not represented because they constituted a very small portion of the total analyzed area.

The landscape configuration in proximity to the study home gardens (25 km^2^ surface area) differs according to the surrounding areas (Fig. 4a, b). Home gardens from the Northern study area are included in the largest urban area, but at the same time are surrounded by large areas of non-fragmented native forest. The home gardens in the center study area are located within a homogeneous and monospecific landscape, due to a high proportion of non-fragmented tree crops cover. Native forest is very fragmented compared to the North and South areas, which may be due to the presence of tree crops and annual crops. The Southern area displays a more heterogeneous landscape. The home gardens within this area appear to be surrounded by the most transformed landscape, the area having been very fragmented and transformed into different productive units.

### Relationship Between Richness, Status, and Landscape Variables

**Fig. 5:**
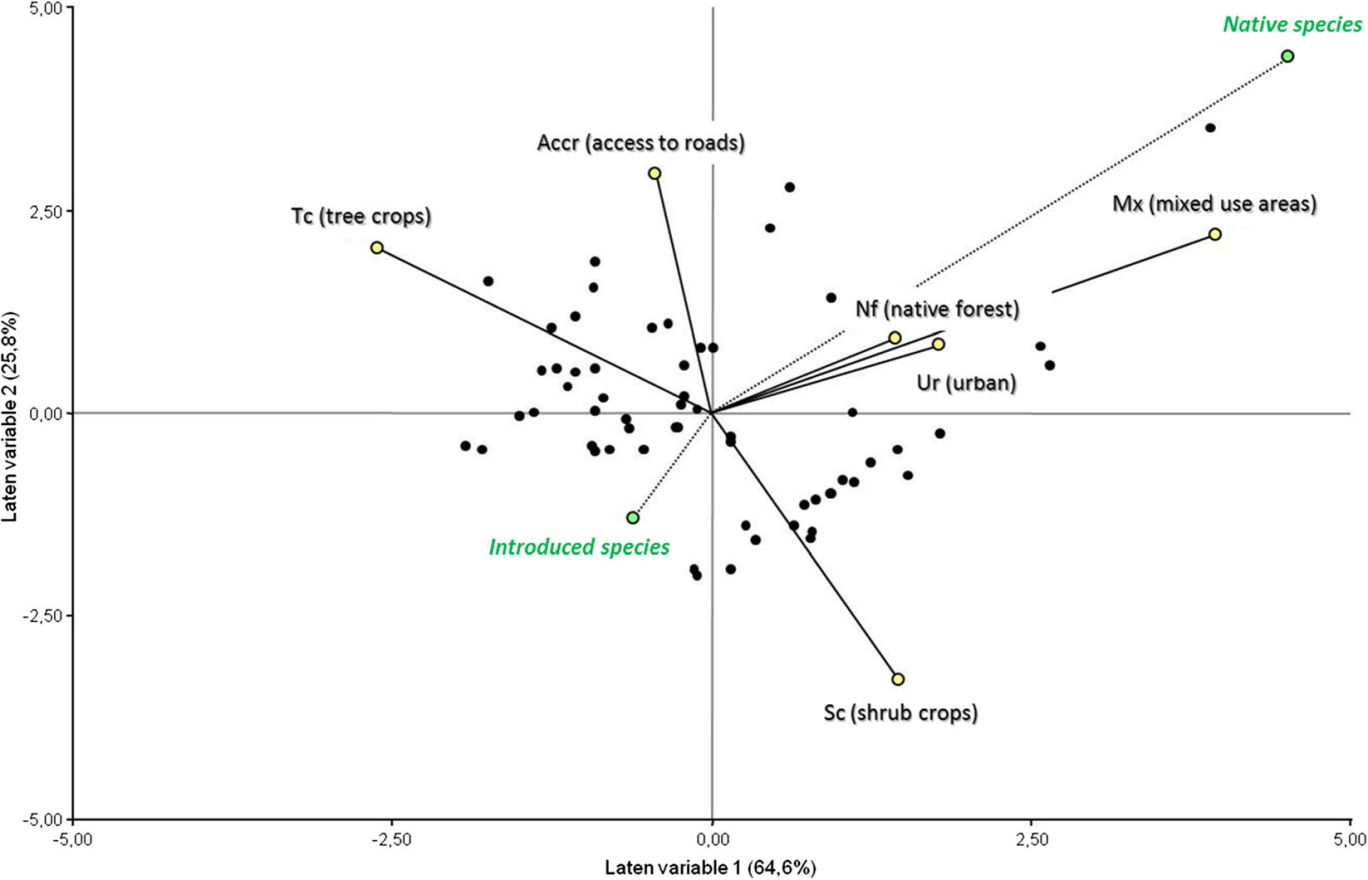
Tri-plot of the first two latent variables obtained in the partial least squares analysis (PLS) performed with the two dependent variables: richness of native and introduced medicinal species (green circles) from 60 Paraguayan home gardens (black circles). The dotted vectors (dependent variables) indicate the magnitude (length) and direction of the change of the predictor variables distance to different land uses and access to national and/or provincial routes and secondary paved roads.

The PLS analysis shows that the first two latent variables explain 90.4% of the variation of the richness of native and introduced species of medicinal plants in the home gardens of Paraguayan immigrants in Misiones (Fig. 5). The first latent variable (Y axis) explains 64.6% of the variation. It is negatively associated with distance to the monocultures of tree crops (Tc) (*R*^2^ = 0.53; *F* = 64.68; *P* ˂ 0.001). It has a positive association with the distance to two land cover classes: mixed use areas (Mx) (*R*^2^ = 0.42; *F* = 41.70; *P* ˂ 0.001) and shrub crops (Sc) (*R*^2^ = 0.35; *F* = 30.56; *P* ˂ 0.001).

The second latent variable (X axis) comprises 25.8% of the variation and is associated negatively with shrub crops (Sc) (*R*^2^ = 0.42; *F* = 42.16; *P* ˂ 0.001), and positively to the distance to the routes and roads (*R*^2^ = 0.42; *F* = 41.46; *P* ˂ 0.001) and to a lesser extent positively to the mixed use areas (Mx) (*R*^2^ = 0.19; *F* = 13.30; *P* = 0.001). The observed trend suggests that gardens closer to shrub crops (Sc) have more introduced species than those gardens further away, whereas introduced species tend to increase as the gardens drift away from the roads. Finally, the first variable is positively and significantly associated with the native species vector (NF) although with a weak R-squared value (*R*^2^ = 0.18; *F* = 13.08; *P* = 0.001).

### Management and Domestication of Medicinal Plants in Paraguayans’ Home Gardens

Different methods of plant management were observed and registered in this study. They can be divided into two kinds: those requiring initial management and those that do not require planting. Within the first group of plants are those that need to be planted through cuttings and other plant propagules, directly transplanted from other habitats, or sown from seeds. These planted species account for 81% of all taxa identified in the home gardens. The planted, sown, and transplanted from other habitats species may become cultivated—and the further activity of a gardener is required, such as watering, weeding, providing shade or sun exposition, or creating barriers for domestic animals. Cultivated plants account for 69% of all medicinal species found in the home gardens of this study. The second group of plants in home gardens are protected species (19%). These plants do not require any initial management, they are sown by animals or the wind, or are plants of early succession, and therefore, it is the decision of a particular gardener to protect them or even convert them into a cultivated species—by providing further tending. Protected species are mainly natives. There are some overlaps between these two groups of cultivated and protected plants—as some species are cultivated by some and just protected by others. The full scope of management of 136 recorded species is presented in Appendix 1, ESM.

## Discussion

### Landscape Structure Affects Medicinal Plant Diversity in Home Gardens

The results show an under-studied relationship between home gardens and the surrounding landscape, and provide novel findings to disciplines that have until now been only loosely related, i.e., ethnobiology and landscape ecology. In our study, an assumption of relationships between land use and biodiversity has been demonstrated and extended to the domestic sphere. Moreover, we contributed evidence to support the environmental scarcity compensation hypothesis. This operates in the following way in the medicinal context; gardeners who live in the proximity of native vegetation have no need to cultivate and protect these resources in their home gardens because they have easy access to them in the nearby landscape.

As we could demonstrate, native species are better represented in gardens situated farther from mixed use areas and are less represented in gardens found farther from tree crops. This remains in agreement with the first prediction that a greater number of native species can be found in home gardens next to tree crops. Gardeners who live in relative proximity to monoculture forest plantations of pine or eucalyptus, and are inserted in a less heterogeneous landscape, have lesser access to wild medicinal resources. Therefore, their gardening efforts are concentrated on planting, transplanting, sowing and protecting native medicinal plants that cannot be found easily in their surroundings. Previous research in our study area reported that 30% of medicinal species used in herbal mixtures with honey by mestizo people and Polish migrants originated from habitats characteristic of mixed use vegetation, being superior to the medicinal species obtained from the native forest (19%) (Kujawska et al. 2012). This finding reinforces the relevance of secondary vegetation in the provision of medicinal resources (see Gavin 2009), and thus explains the relatively weak relationship between the native forest and the richness of medicinal plants in our analysis (Fig. 5).

On the other hand, variable distance to shrub crops and to roads contributes to the explanation of the presence of introduced species, although these are not correlated to the second latent variable of the model. Therefore, the richness of introduced species in home gardens would not be fully explained by landscape variables associated with the availability of medicinal resources. At this point, we assume that social webs of exchange of propagules and seeds may have prevalence over landscape factors (see Díaz-Reviriego et al. 2016). However, this aspect has not been studied in this research.

Although with some exceptions (Peroni et al. 2016), a considerable number of studies have reported greater biodiversity richness in peri-urban and urban home gardens than in those situated farther from urban centers (Bernholt et al. 2009; Heckler 2007; WinklerPrins 2002). This was explained by a greater possibility of exchange of knowledge and plant material among the home gardeners. However, those studies did not take into account the possible effect of landscape on plant availability and therefore access to these resources (see Gaoue et al. 2017). On the other hand, Wezel and Ohl (2005) explain that the daily travel of farmers to swiddens and cultivated plots may reduce the need for diverse home gardens in rural areas. Larios et al. (2013) found that the most diverse Nahuatl home gardens were located in areas with less diverse vegetation, suggesting the importance of the scarcity compensation effect. Similar results were found by Poot-Pool and collaborators (2015), who observed that people also tend to grow medicinal resources, which are not directly available in their surrounding landscape. Although these studies contribute to understanding of the influence of the landscape on the diversity of home gardens, our results are the first to provide conclusive data in favor of the scarcity compensation hypothesis on the diversity of medicinal plants. While the predominant view is that home gardens emulate natural vegetation in their interior, as models of agroforestry systems (Larios et al. 2013; Peroni et al. 2016), according to our results home gardens can be considered as a complement to what is not available in the environment.

These findings provide new arguments in the ethnobotanical debate concerning relevant hypotheses, such as the hypothesis of ecological appearance or the plant use value hypothesis, among others (Albuquerque 2006; Gaoue et al. 2017; Gonçalves et al. 2016). Our results indicate that less abundant resources in the environment are not necessarily less used, because a portion of these resources, probably intensely used ones, are transferred to home gardens where they are readily available for use. Thus, the hypothesis of ecological appearance makes sense when it takes into account species occurring naturally in the landscape as well as tended in home gardens. As we could observe, the landscape accessibility of wild medicinal resources motivates management practices that will finally affect the use patterns of the species, regardless of whether they are available or not in the vicinity of the family residence.

### The Double Role of Home Gardens: the Domestication of Native Species and Acclimatization of Introduced Ones

The richness of native species in home gardens outnumbered the introduced ones. The diversified management practices in Paraguayans’ home gardens show another facet of human-plant relatedness, as well as the interaction between home gardens and the surrounding vegetation. Wild native plants that invade gardens are not removed by gardeners when they are considered useful. Other species are transplanted from natural habitats, planted from vegetative propagules and sown from seeds, especially when gardeners observe their absence in their immediate surroundings. These practices have been observed in other studies as well (Blanckaert et al. 2004; Coomes 2010; Vogl-Lukasser and Vogl 2010). Hence, home gardens become valuable sites for researching the domestication of plant populations (Casas et al. 1996).

Of all the registered species in our study, fruit trees from the Myrtaceae family, such as *Eugenia uniflora* L., *Eugenia involucrata* DC., and *Eugenia pyriformis* Cambess. represent canonical cases of plants that are in the process of domestication. This finding remains in agreement with Clement’s (1999) affirmation about *E. uniflora*, but here it has been broadened to others species of Myrtaceae. However, it can be presumed that the domestication of these particular species is enhanced by their double role as edible and medicinal resources (Paraguayan people commonly utilize leaves as medicine and fruit as snacks). Other incipiently domesticated plants analyzed by Clement, such as *Garcinia brasiliensis* Mart. and *Acrocomia aculeata* (Jacq.) Lodd. ex Mart. were also found in Paraguayans’ home gardens. Incipiently domesticated plants are those at an early stage of domestication with relatively low phenotypic and genetic differentiation compared to their wild relatives (Lins Neto et al. 2014).

Home gardens may also be perceived as places where some introduced species may potentially become invasive after a process of adaptation/acclimation prior to invasion. Practices in home gardens not only can reduce risk of plant failure due to protection from unsuitable environmental conditions and sufficient time for them to adapt to local conditions, but also, because they can regulate the location of seed sources and increase the propagule pressure (Hulme 2011; Mack 2000; Pergl et al. 2016). The importance of several introduced plants in Misiones is closely related to the history of this multi-cultural region (Bartolomé and Schiavoni 2008; Kujawska and Pardo de Santayana 2015). Overall, although several plants of foreign origin are important medicinal resources in the home gardens of Paraguayan migrants, this fact does not contradict the role of the Paraguayan diaspora in the promotion and protection of native medicinal resources. Other studies document that native species richness was sustained with the introduction of species (Akinnifesi et al. 2010). Here, we show that the home gardens of Paraguayan migrants display the double function of intensive cultivation and incipient management of several native plant resources. Moreover, the prevalence of native species in home gardens suggest that Paraguayan people who hail from the ecoregion of the Atlantic Forest in Paraguay can continue their phytotherapy in an undisturbed way in Misiones. In these circumstances, knowledge on native plants is transmitted and exercised in the host country. The assessment of these practices and the importance of native species for the preservation of the natural environment in Misiones, has been, however, beyond the range of our research.

## Conclusions

The presented approach in research on medicinal plant richness in home gardens is a breakthrough in the study of home gardens. We outlined a novel methodology, which combines approaches and tools from ethnobiology and landscape ecology. We applied coarse grain and fine grain analysis, which enabled us to establish a relationship between the characteristic of the landscape and the plant diversity in home gardens. We contributed evidence in support of the environmental scarcity compensation hypothesis. We identified the effect of landscape variables on the richness of native species, suggesting that access to, and availability of, these resources influence the decisions of Paraguayan immigrants regarding what is preserved and managed in the gardens. Finally, it is important to point out the possible role of home gardens as social spaces where native plants are preserved, and where the process of plant domestication is most likely to occur.

## Electronic supplementary material

ESM 1 (DOC 219 kb)
