# Supplementary material for: Effects of Landscape Structure on Medicinal Plant Richness in Home Gardens: Evidence for the Environmental Scarcity Compensation Hypothesis
Source: Econ Bot. 2018 May 30;72(2):150–65. doi: 10.1007/s12231-018-9417-3 (PMC6182649; doi:10.1007/s12231-018-9417-3)
Supplement: Supplementary file 1 — (DOC 219 kb) [file 12231_2018_9417_MOESM1_ESM.doc]

Appendix 1. Electronic Supplementary Material, ESM. Medicinal plant species grown in home gardens of Paraguayan immigrants in Misiones, Argentina.

| **Species name** | **Local name** | **Origin** | **Nr of home gardens** | **Initial* management** | | | | **Further management*** | |
| --- | --- | --- | --- | --- | --- | --- | --- | --- | --- |
|  |  |  |  | **P** | **S** | **T** | **N** | **C** | **Pr.** |
| *Acanthospermum australe* (Loefl.) Kuntze | tapekue | native | 5 |  |  |  | 5 |  | 5 |
| *Achillea millefolium* L. | milenramas | introduced | 2 |  | 2 |  |  | 2 |  |
| *Achyrocline alata* (Kunth) DC., *Achyrocline satureioides*(Lam.) DC. | marcela, jate'i ka'a | native | 5 |  | 2 |  | 3 | 1 | 4 |
| *Acrocomia aculeata* (Jacq.) Lodd. ex Mart. | coco | native | 1 |  |  |  | 1 |  | 1 |
| *Allium sativum* L. | ajo | introduced | 4 | 4 |  |  |  | 4 |  |
| *Allophylus edulis* (A.St.-Hil., A.Juss. & Cambess.) Radlk. | kokû | native | 18 | 7 |  |  | 11 | 4 | 14 |
| *Aloe arborescens* Mill. | aloe de hoja fina | introduced | 10 | 10 |  |  |  | 10 |  |
| *Aloe maculata* All. | aloe de hoja ancha | introduced | 26 | 26 |  |  |  | 26 |  |
| *Aloysia citriodora* Palau | cedrón del palo | native | 2 | 2 |  |  |  | 2 |  |
| *Aloysia gratissim*a (Gillies & Hook.) Tronc. | poleo en palo | native | 1 | 1 |  |  |  | 1 |  |
| *Aloysia polystachya* (Griseb.) Moldenke | burrito | native | 11 | 11 |  |  |  | 11 |  |
| *Aloysia pulchra* (Briq.) Moldenke | niño rupa, poleo | native | 2 | 2 |  |  |  | 2 |  |
| *Alternanthera brasiliana* (L.) Kuntze | penicilina | native | 12 | 12 |  |  |  | 9 | 3 |
| *Alternanthera pungens* Kunth | yerba del pollo | native | 2 |  |  |  | 2 |  | 2 |
| *Ambrosia artemisifolia* L. | altamisa | native | 3 |  | 1 |  | 2 | 1 | 2 |
| *Anethum graveolens* L. | eneldo | introduced | 3 |  | 3 |  |  | 3 |  |
| *Argemone subfusiformis* Ownbey | cardo santo | native | 3 | 3 |  |  |  | 3 |  |
| *Aristolochia triangularis* Cham. | ysypo milhombres | native | 4 |  |  | 4 |  | 2 | 2 |
| *Artemisia absinthium* L. | ajenjo | introduced | 22 | 22 |  |  |  | 22 |  |
| *Artemisia alba* Turra | alcanfor en hoja | introduced | 7 | 7 |  |  |  | 7 |  |
| *Baccharis gaudichaudiana* DC. | chirca melosa | native | 3 | 3 |  |  |  | 3 |  |
| *Baccharis trimera* (Less.) DC. | jaguarete ka'a, carqueja | native | 14 | 14 |  |  |  | 12 | 2 |
| *Bauhinia forficata* Link | pata de buey | native | 4 | 1 |  | 2 | 1 | 1 | 3 |
| *Bauhinia microstachya* (Raddi) J.F.Macbr. | kai escalera, pata de buey i | native | 4 | 1 |  | 2 | 1 | 1 | 3 |
| Begonia cucullata Willd. | agrial | native | 4 |  |  | 2 | 2 | 2 | 2 |
| *Bidens pilosa* L., *Bidens subalternans* DC. | amor seco, picón | native | 2 |  |  |  | 2 |  | 2 |
| *Boerhavia diffusa* L. | ka'arurupe | introduced | 2 |  |  |  | 2 |  | 2 |
| *Cajanus cajan* (L.) Millsp. | kumanda yvyra'i | introduced | 3 |  | 3 |  |  | 3 |  |
| *Calendula officinalis L.* | calendula | introduced | 1 |  | 1 |  |  | 1 |  |
| *Calyptocarpus biaristatus (DC.) H. Rob.* | pato rupa | native | 1 |  |  |  | 1 |  | 1 |
| *Campomanesia xanthocarpa* (Mart.) O.Berg | guavira | native | 5 | 2 |  | 1 | 2 | 1 | 4 |
| *Carica papaya* L. | mamón | naturalized | 6 |  | 4 |  | 2 | 1 | 5 |
| *Cecropia pachystachya* Trécul | amba'y | native | 3 | 1 |  |  | 2 |  | 3 |
| *Celosia argentea* L. | penacho | naturalized | 2 |  | 2 |  |  | 2 |  |
| *Cissus verticillata* (L.) Nicolson & C.E.Jarvis | insulina | native | 2 | 2 |  |  |  | 2 |  |
| *Citrus* x *aurantium* L. | apepu | naturalized | 6 | 6 |  |  |  | 6 |  |
| *Citrus limon (L.) Osbeck* | limón | introduced | 5 | 5 |  |  |  | 5 |  |
| *Citrus paradisi* Macfad. | pomelo | introduced | 1 | 1 |  |  |  | 1 |  |
| *Citrus reticulata* Blanco | mandarina | introduced | 3 | 3 |  |  |  | 3 |  |
| *Citrus sinensis* (L.) Osbeck | naranja | introduced | 11 | 11 |  |  |  | 11 |  |
| *Cordia ecalyculata* Vell. | horquetero, colita | native | 1 |  |  |  | 1 |  | 1 |
| *Coriandrum sativum*L. | kuratû | introduced | 7 |  | 7 |  |  | 6 | 1 |
| *Costus* sp. | caña brava | introduced | 3 | 3 |  |  |  | 3 |  |
| *Croton* sp. | sangre de grado | native | 2 |  |  | 1 | 1 |  | 2 |
| *Cuphea glutinosa* Cham.& Schltdl. | siete sangrías | native | 2 | 2 |  |  |  | 2 |  |
| *Cyclospermum leptophyllum* (Pers.) Sprague | apio i | native | 2 |  |  |  | 2 |  | 2 |
| *Cymbopogon citratus* (DC.) Stapf | cedrón | introduced | 24 | 24 |  |  |  | 24 |  |
| *Cynodon dactylon* (L.) Pers. | gramilla | introduced | 1 |  |  |  | 1 |  | 1 |
| *Dioscorea* sp. | papa al aire | introduced | 1 | 1 |  |  |  | 1 |  |
| *Dolichandra unguis-cati* (L.) L.G. Lohmann | uña del gato | native | 4 | 2 |  |  | 2 | 2 | 2 |
| *Dorstenia brasiliensi*s Lam. | tarope | native | 2 |  |  | 2 |  | 2 |  |
| *Dysphania ambrosioides* (L.) Mosyakin & Clemants | ka'arê | introduced | 11 |  | 1 |  | 10 |  | 11 |
| *Eichhornia crassipes* (Mart.) Solms | camalote | native | 1 |  |  | 1 |  | 1 |  |
| *Elionurus muticus* (Spreng.) Kuntze | kapi'cedrón | native | 3 | 3 |  |  |  | 3 |  |
| *Equisetum giganteum* L. | cola de caballo | native | 1 | 1 |  |  |  | 1 |  |
| *Eriobotrya japonica* (Thunb.) Lindl. | níspero | introduced | 2 | 1 |  |  | 1 |  | 2 |
| *Eugenia involucrata* DC. | cerella | native | 1 | 1 |  |  |  | 1 |  |
| *Eugenia pyriformis* Cambess. | yva hâi | native | 3 | 2 |  |  | 1 | 2 | 1 |
| *Eugenia uniflora* L. | ñangapiry, pitanga | native | 12 | 6 | 3 |  | 3 | 5 | 7 |
| *Eupatorium inulifolium* (Kunth) Hieron. | doctorcito | native | 2 |  |  |  | 2 |  | 2 |
| *Euphorbia prostrata* Aiton | tupasy kamby | native | 1 |  |  |  | 1 |  | 1 |
| *Foeniculum vulgare* Mill. | hinojo | introduced | 6 |  | 6 |  |  | 6 |  |
| *Garcinia brasiliensis* Mart. | pakuri | native | 1 | 1 |  |  |  | 1 |  |
| *Gleditsia amorphoides* (Griseb.) Taub. | espina de corona | native | 1 |  |  |  | 1 |  | 1 |
| *Gomphrena celosioides* Mart. | perudilla | native | 2 |  |  |  | 2 |  | 2 |
| *Gomphrena perennis* L. | siempre viva | native | 4 |  | 4 |  |  | 4 |  |
| *Hemionitis tomentosa* (Lam.) Raddi | doradilla | native | 3 |  |  | 3 |  | 3 |  |
| *Heteropterys glabra* Hook & Arn. | tilo | native | 11 | 11 |  |  |  | 9 | 2 |
| *Hypochaeris chillensis* (Kunth) Hieron. | achicoria silvestre | native | 5 |  | 1 |  | 4 |  | 5 |
| *Jacaranda micrantha* Cham. | karoba | native | 1 |  |  | 1 |  |  | 1 |
| *Lactuca virosa* Habl. | lechuga japonesa | introduced | 1 |  |  |  | 1 |  | 1 |
| *Lavandula* sp. | alucema | introduced | 2 | 2 |  |  |  | 2 |  |
| *Lepidium didymum* L. | mentruz | introduced | 3 |  |  |  | 3 |  | 3 |
| *Lippia alba* (Mill.) N.E.Br. ex Britton & P.Wilson | salvia | native | 32 | 32 |  |  |  | 31 | 1 |
| *Lippia brasiliensis* (Link) T.R.S. Silva | jate'i ka'a ka'aguy | native | 5 | 4 |  | 1 |  | 5 |  |
| *Luehea divaricata* Mart. | sotacaballo | native | 1 |  |  |  | 1 |  | 1 |
| *Malva sylvestris* L. | malva de castilla | introduced | 2 | 2 |  |  |  | 1 | 1 |
| *Mangifera indica* L. | mango | introduced | 2 | 2 |  |  |  | 2 |  |
| *Matricaria chamomilla* L. | manzanilla | introduced | 13 |  | 4 |  | 9 | 4 | 9 |
| *Maytenus ilicifolia* Mart. ex Reissek | kangorosa | native | 15 | 6 | 1 | 6 | 2 | 8 | 7 |
| *Melilotus* sp. | alfalfa | introduced | 2 |  | 2 |  |  | 2 |  |
| *Melissa officinalis* L. | toronjíl, melisa | introduced | 7 | 7 |  |  |  | 7 |  |
| *Mentha* spp. | menta, menta poleo, yerba buena | introduced | 23 | 23 |  |  |  | 23 |  |
| *Moringa oleifera* Lam. | moringa | introduced | 6 | 5 | 1 |  |  | 6 |  |
| *Morus alba* L. | mora | introduced | 1 | 1 |  |  |  | 1 |  |
| *Muehlenbeckia sagittifolia* (Ortega) Meisn. | zarzaparilla | native | 1 | 1 |  |  |  | 1 |  |
| *Myrocarpus frondosus* Allemao | insienso | native | 1 |  |  | 1 |  |  | 1 |
| *Nicotiana glauca* Graham | palan palan | native | 1 | 1 |  |  |  |  | 1 |
| *Ocimum basilicum* L. | albahaca | introduced | 2 | 1 | 1 |  |  | 2 |  |
| *Ocimum* cf. *basilicum* var. *anisatum*  Benth. | anís | introduced | 9 | 9 |  |  |  | 9 |  |
| *Opuntia* spp. | tuna | native | 1 | 1 |  |  |  | 1 |  |
| *Origanum vulgare* L. | orégano | introduced | 7 | 5 | 2 |  |  | 7 |  |
| *Panicum tricholaenoides* Steud. | cola de caballo | native | 2 | 2 |  |  |  | 2 |  |
| *Parietaria debilis* G. Forst. | ka'a piky | native | 1 |  |  |  | 1 |  | 1 |
| *Passiflora alata* Curtis | mburukuja | native | 3 | 3 |  |  |  | 3 |  |
| *Peperomia circinnata* Link | jatevu ka'a | native | 1 |  |  | 1 |  |  | 1 |
| *Persea americana* Mill. | palta | naturalized | 3 | 2 |  |  | 1 | 1 | 2 |
| *Petiveria alliacea* L. | pipi | native | 8 | 4 | 1 | 2 | 1 | 3 | 5 |
| *Petroselinum crispum* (Mill.) Fuss | perejíl | introduced | 1 |  | 1 |  |  | 1 |  |
| *Philodendron bipinnatifidum* Schott ex Endl. | guembe | native | 2 |  |  | 2 |  |  | 2 |
| *Phyllanthus niruri* L. | quebrapiedras, rompepiedras | native | 5 | 1 |  |  | 4 | 1 | 4 |
| *Picrasma crenata* Engl. in Engl. & Prantl | palo amargo | native | 1 |  |  | 1 |  | 1 |  |
| *Piper mikanianum* (Kunth) Steud. | pariparoba | native | 4 | 3 |  | 1 |  | 3 | 1 |
| *Plantago australis* Lam. | llantén | native | 9 |  | 1 |  | 8 |  | 9 |
| *Plectranthus* sp. | boldo | introduced | 5 | 5 |  |  |  | 5 |  |
| *Plinia involucrata* (O.Berg) McVaugh | cerella | native | 1 | 1 |  |  |  | 1 |  |
| *Plinia peruviana* (Poir.) Govaerts | yaboticaba | native | 2 | 2 |  |  |  | 2 |  |
| *Pluchea sagittalis* Less. | yerba del lucero | native | 2 |  |  |  | 2 |  | 2 |
| *Portulaca oleracea* L. | verdolaga | introduced | 1 |  |  |  | 1 |  | 1 |
| *Prunus persica* (L.) Batsch | durazno | introduced | 1 | 1 |  |  |  | 1 |  |
| *Psidium* spp. | guayaba, arasa | native | 6 | 4 |  |  | 2 | 3 | 3 |
| *Punica granatum* L. | granada | introduced | 3 | 3 |  |  |  | 3 |  |
| *Rhipsalis cereuscula Haw.* | suelta con suelta | native | 2 | 2 |  |  |  |  | 2 |
| *Rollinia salicifolia* Schltdl. | aratiku | native | 8 | 4 | 3 |  | 1 | 3 | 5 |
| *Rosa* sp. | rosa blanca | introduced | 7 | 7 |  |  |  | 7 |  |
| *Rosmarinus officinalis* L. | romero | introduced | 23 | 23 |  |  |  | 23 |  |
| *Ruta chalepensis* L. | ruda | introduced | 24 | 24 |  |  |  | 24 |  |
| *Sambucus australis* Cham. & Schltdl. | sauco | native | 2 | 1 |  | 1 |  | 1 | 1 |
| *Scoparia dulcis* L. | typycha kuratû | native | 2 |  | 1 |  | 1 |  | 2 |
| *Sida cordifolia* L. | malva blanca | native | 11 |  | 4 |  | 7 |  | 11 |
| *Smallanthus connatus* (Spreng.) H.Rob. | jaguarete po | native | 4 | 4 |  |  |  | 4 |  |
| *Solanum americanum* Mill. | arechichu | native | 2 |  |  |  | 2 |  | 2 |
| *Solidago chilensis* Meyen | teju ka'a | native | 3 |  | 3 |  |  | 2 | 1 |
| *Stachytarpheta cayennensis* (Rich.) Vahl | tatu ruguái | native | 1 |  |  |  | 1 |  | 1 |
| *Stevia rebaudiana* (Bertoni) Bertoni | ka'a he'ê | native | 1 | 1 |  |  |  | 1 |  |
| *Tagetes minuta* L. | suico | native | 5 |  | 3 |  | 2 | 2 | 3 |
| *Tanacetum parthenium* (L.) Sch. Bip. | manzanilla guasu | introduced | 1 |  | 1 |  |  | 1 |  |
| *Tanacetum vulgare* L. | palmita | introduced | 2 | 2 |  |  |  | 2 |  |
| *Taraxacum* spp. | diente de león | introduced | 1 |  |  |  | 1 |  | 1 |
| *Urera baccifera* (L.) Gaudich. ex Wedd. | ortiga grande | native | 1 |  |  | 1 |  |  | 1 |
| *Urtica circularis* Sorarú | ortiga chica | native | 1 |  |  |  | 1 |  | 1 |
| *Verbena litoralis* Kunth, Verbena montevidensis Spreng. | vervena | native | 7 |  |  |  | 7 |  | 7 |
| *Vernonia amygdalina* Delile | yurubeba | introduced | 1 | 1 |  |  |  | 1 |  |
| *Viola* sp. | violeta | introduced | 2 | 2 |  |  |  | 2 |  |
| *Vitex megapotamica* (Spreng.) Moldenke | taruma | native | 2 | 1 |  |  | 1 | 1 | 1 |
| *Xanthium spinosum* L. | cepacaballo, abrojito | introduced | 6 |  | 5 |  | 1 | 3 | 3 |

* Initial management: P – planted, S – sown, T – transplanted from the forest, N – none initial management; Further management: C – cultivated, Pr. - protected
